# Supplementary material for: Characterization of the DNA accessibility of chloroplast genomes in grasses
Source: Commun Biol. 2024 Jun 22;7:760. doi: 10.1038/s42003-024-06374-4 (PMC11193712; doi:10.1038/s42003-024-06374-4)
Supplement: Supplementary file 2 — Supplementary Information [file 42003_2024_6374_MOESM2_ESM.pdf]

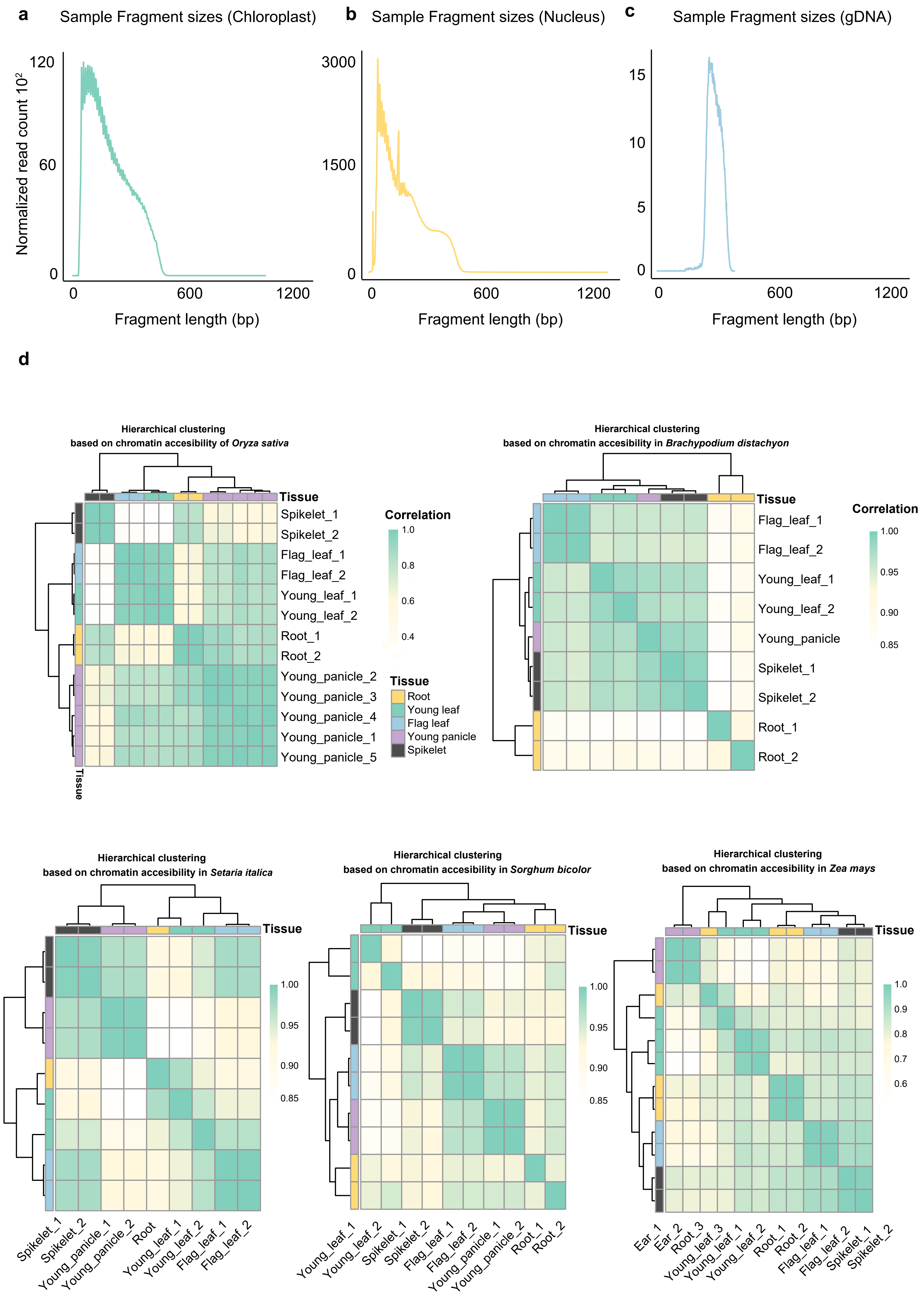

a
Sample Fragment sizes (Chloroplast)
b
Sample Fragment sizes (Nucleus)
c
Sample Fragment sizes (gDNA)
Normalized read count 10^2
Fragment length (bp)
d
Hierarchical clustering
based on chromatin accesibility of Oryza sativa
Tissue
Spikelet_1
Spikelet_2
Flag_leaf_1
Flag_leaf_2
Young_leaf_1
Young_leaf_2
Root_1
Root_2
Young_panicle_2
Young_panicle_3
Young_panicle_4
Young_panicle_1
Young_panicle_5
Correlation
Tissue
Root
Young leaf
Flag leaf
Young panicle
Spikelet
Hierarchical clustering
based on chromatin accesibility in Brachypodium distachyon
Flag_leaf_1
Flag_leaf_2
Young_leaf_1
Young_leaf_2
Young_panicle
Spikelet_1
Spikelet_2
Root_1
Root_2
Hierarchical clustering
based on chromatin accesibility in Setaria italica
Spikelet_1
Spikelet_2
Young_panicle_1
Young_panicle_2
Root
Young_leaf_1
Young_leaf_2
Flag_leaf_1
Flag_leaf_2
Hierarchical clustering
based on chromatin accesibility in Sorghum bicolor
Young_leaf_1
Young_leaf_2
Spikelet_1
Spikelet_2
Flag_leaf_1
Flag_leaf_2
Young_panicle_1
Young_panicle_2
Root_1
Root_2
Hierarchical clustering
based on chromatin accesibility in Zea mays
Ear_1
Ear_2
Root_3
Young_leaf_3
Young_leaf_1
Young_leaf_2
Root_1
Root_2
Flag_leaf_1
Flag_leaf_2
Spikelet_1
Spikelet_2

Supplementary Figure 1. Quality control of ATAT-seq data. (a) Histogram of insert size of ATAC-seq fragments mapped to the chloroplast genome. ATAC-seq data from rice young leaves (sample: Young_leaf_1) was used. **(b)** Histogram of insert size of ATAC-seq fragments mapped to the nuclear genome. ATAC-seq data from rice young leaves (sample: Young_leaf_1) was used. **(c)** Histogram of insert size of tagmentation fragments of naked gDNA (from rice young leaves) mapped to the chloroplast genome. **(d)** Heatmap of correlations among tissues and replicates for five grasses. We first divided the cpDNA into 100 bp bins and quantified the number of Tn5 insertions in each bin (extended to 150 bp when counting) as the accessibility of each dataset, and then we calculated Pearson correlation coefficients for accessibility between different datasets.

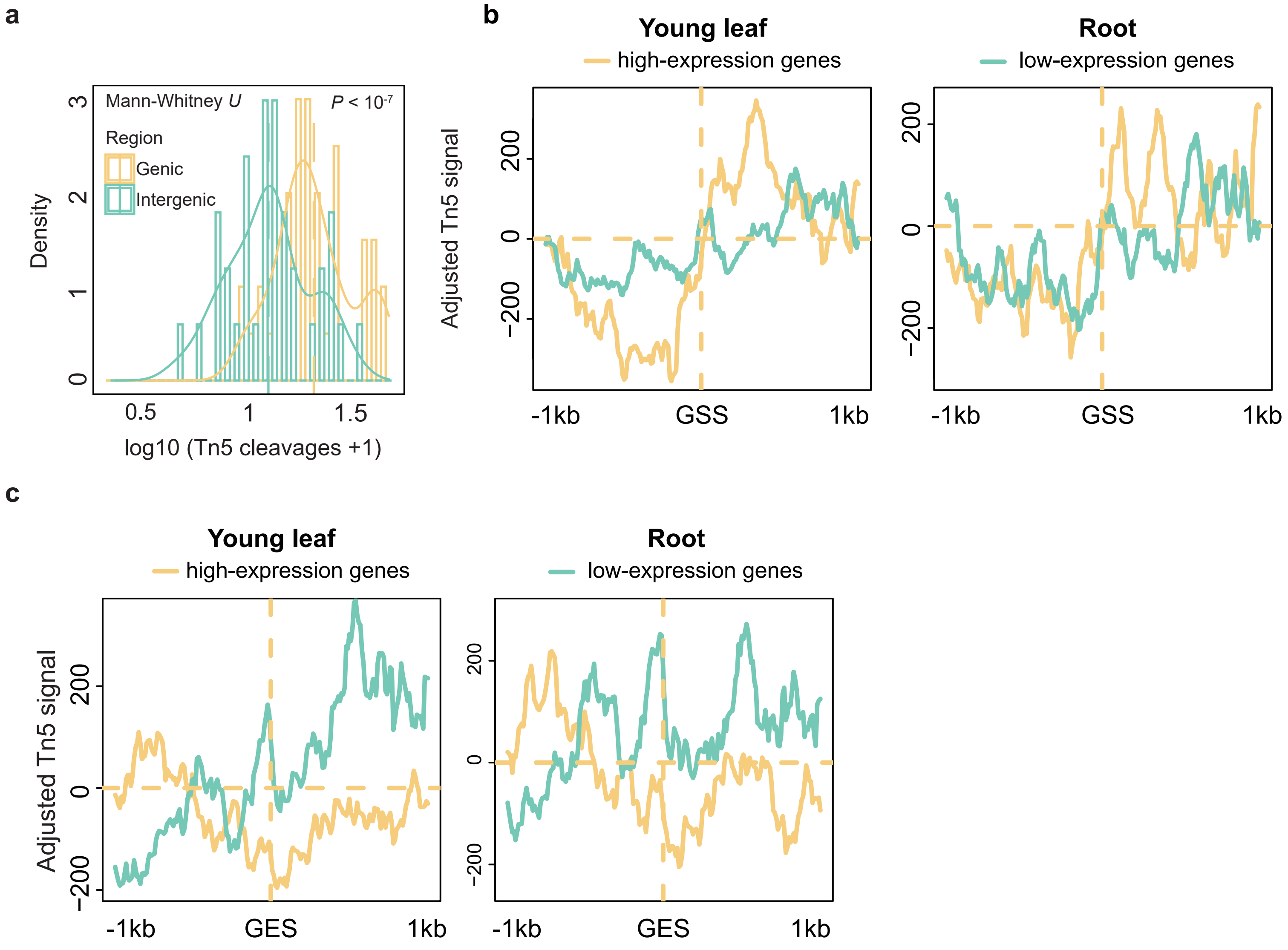

Supplementary Figure 2. Accessibility around the start and end sites of cistrons with different expression level in rice cpDNA. The annotated start site of the first gene of the 43 reported cistrons (Kanno A et al., 1993; Zhelyazkova et al., 2012; Shi et al., 2016) was used as the start site of the cistron, and the annotated end site of the last gene of the cistron was used as the end site of the cistron. **(a)**The distributions of accessibility in genic (43 reported operons) and intergenic regions. The x-axis is the average Tn5 cleavages of per region (first sum the Tn5 cleavages for each genic and intergenic regions, then divided by its length), while y-axis represents the density. **(b)** According to the classification of the expression level of the first gene of cistron, 8 belonged to "high expression" and 14 belonged to "low expression". **(c)** According to the expression level of the last gene of cistron, 11 belonged to "high expression" and 12 belonged to "low expression". The average accessibility of 200 bp at both ends (400 bp in total) was defined as the background and subtracted from the average accessibility at each position for adjustment.

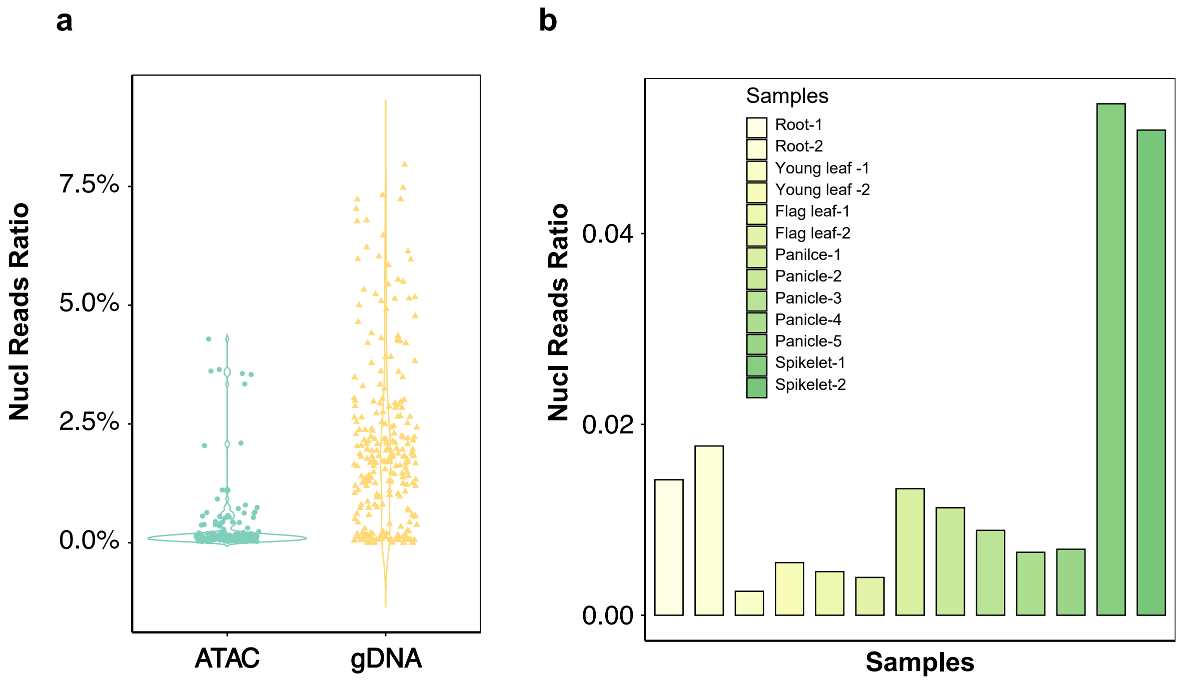

Supplementary Figure 3. Proportion of NUPT reads in reads mapped to cpDNA in rice. **(a)** NUPT ratio in tagmentation of naked gDNA and ATAC samples, respectively. **(b)** NUPT ratio of each ATAC sample.

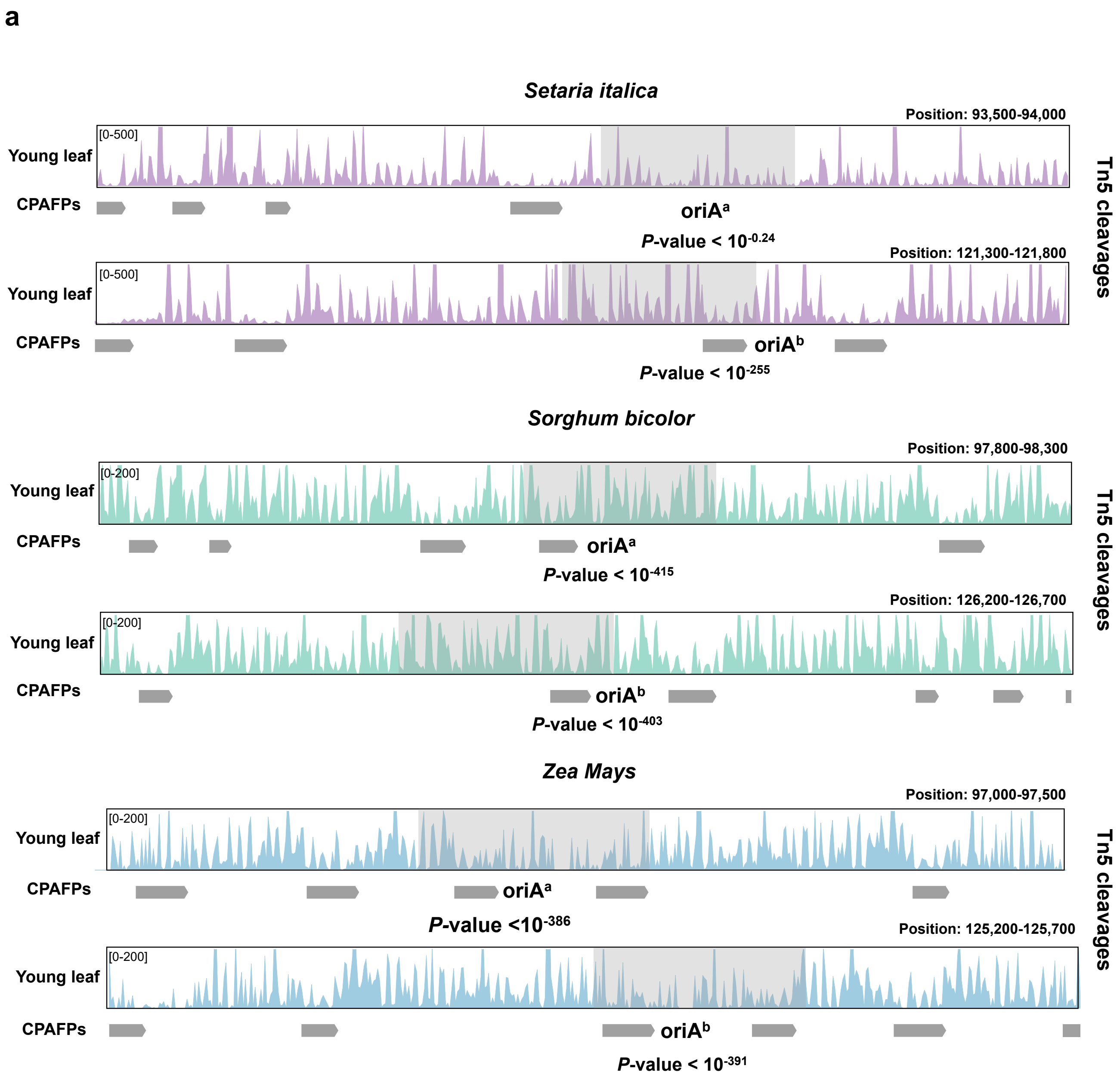

Supplementary Figure 4. Profiles of Tn5 insertions per site and CPAFPs in oriA regions of grasses.

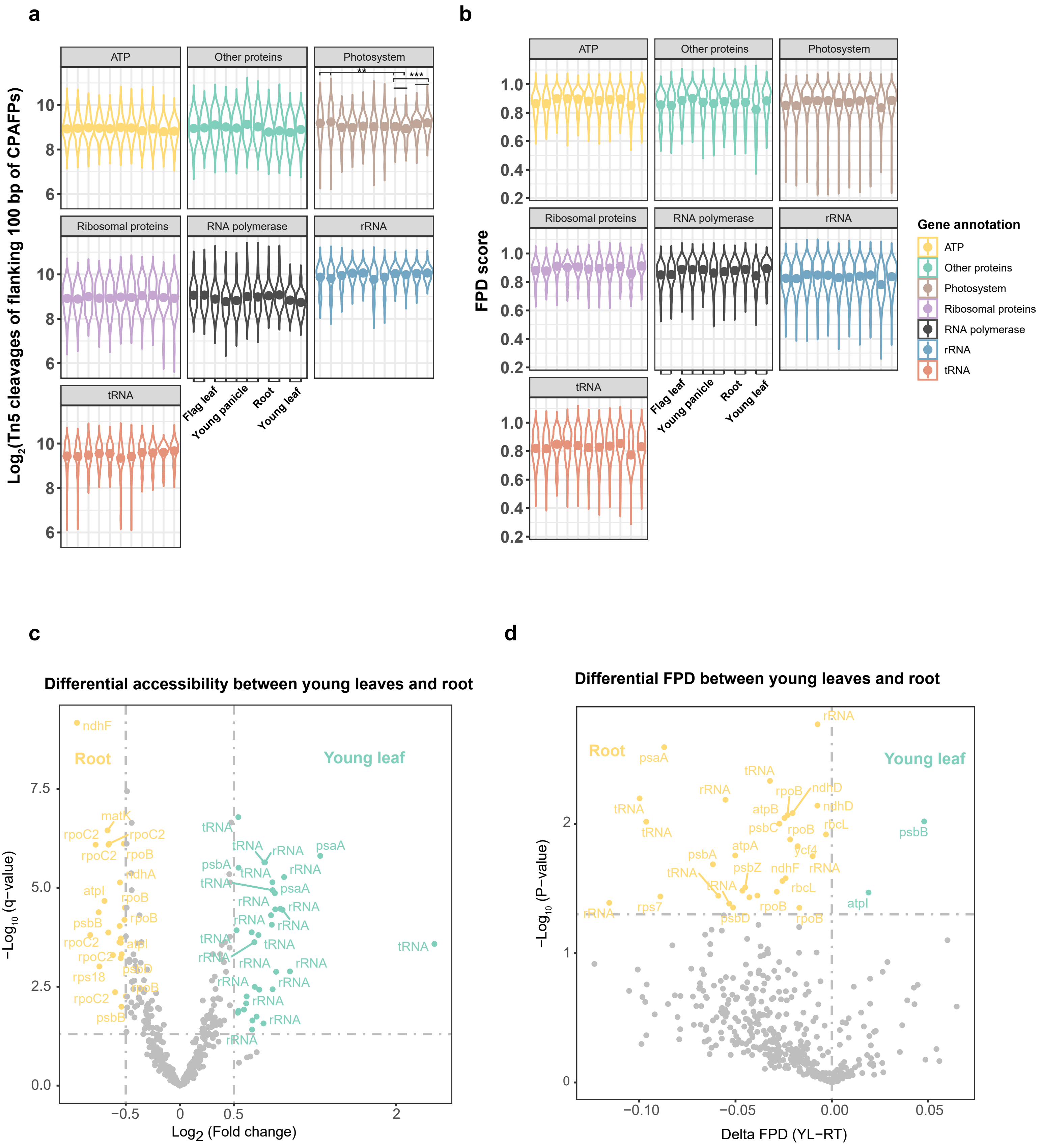

a
Log2(Tn5 cleavages of flanking 100 bp of CPAFPs)
ATP
Other proteins
Photosystem
Ribosomal proteins
RNA polymerase
rRNA
tRNA
**
***
Flag leaf
Young panicle
Root
Young leaf
b
FPD score
Gene annotation
ATP
Other proteins
Photosystem
Ribosomal proteins
RNA polymerase
rRNA
tRNA
c
Differential accessibility between young leaves and root
Root
Young leaf
−Log10 (q−value)
Log2 (Fold change)
d
Differential FPD between young leaves and root
Root
Young leaf
−Log10 (P−value)
Delta FPD (YL−RT)

Supplementary Figure 5. The distributions of FPD scores and flanking accessibility signals of CPAFPs in different tissues. (a) Accessibility of flanking regions of the CPAFPs in the genic regions of genes in different functional categories. The flanking region is defined as a 100 bp region centered at the midpoint of the CPAFP. We selected 421 CPAFPs identified in at least one tissue and located in the genic regions in rice for this analysis. **(b)** FPD scores of CPAFPs. The selected CPAFPs were the same as **a**. **(c)** Differential accessibility analysis of flanking regions of CPAFPs between young leaves and roots. The definition of flanking region is the same as **a**. The fold-change and q-value were calculated by DESeq2. CPAFPs with higher accessibility in young leaves are colored by green [log2 (fold-change) >0.5], while CPAFPs with higher accessibility in roots are colored by yellow [log2 (fold-change) <-0.5]. The selected CPAFPs were the same as **a**. **(d).** Differential FPD analysis of CPAFPs between young leaves and roots. The *P*-value were calculated by paired *t*-test. CPAFPs with higher FPD in young leaves are colored by green (*P*-value < 0.05), while CPAFPs with higher FPD in roots are colored by yellow (*P*-value <0.05). The selected CPAFPs were the same as **a**.

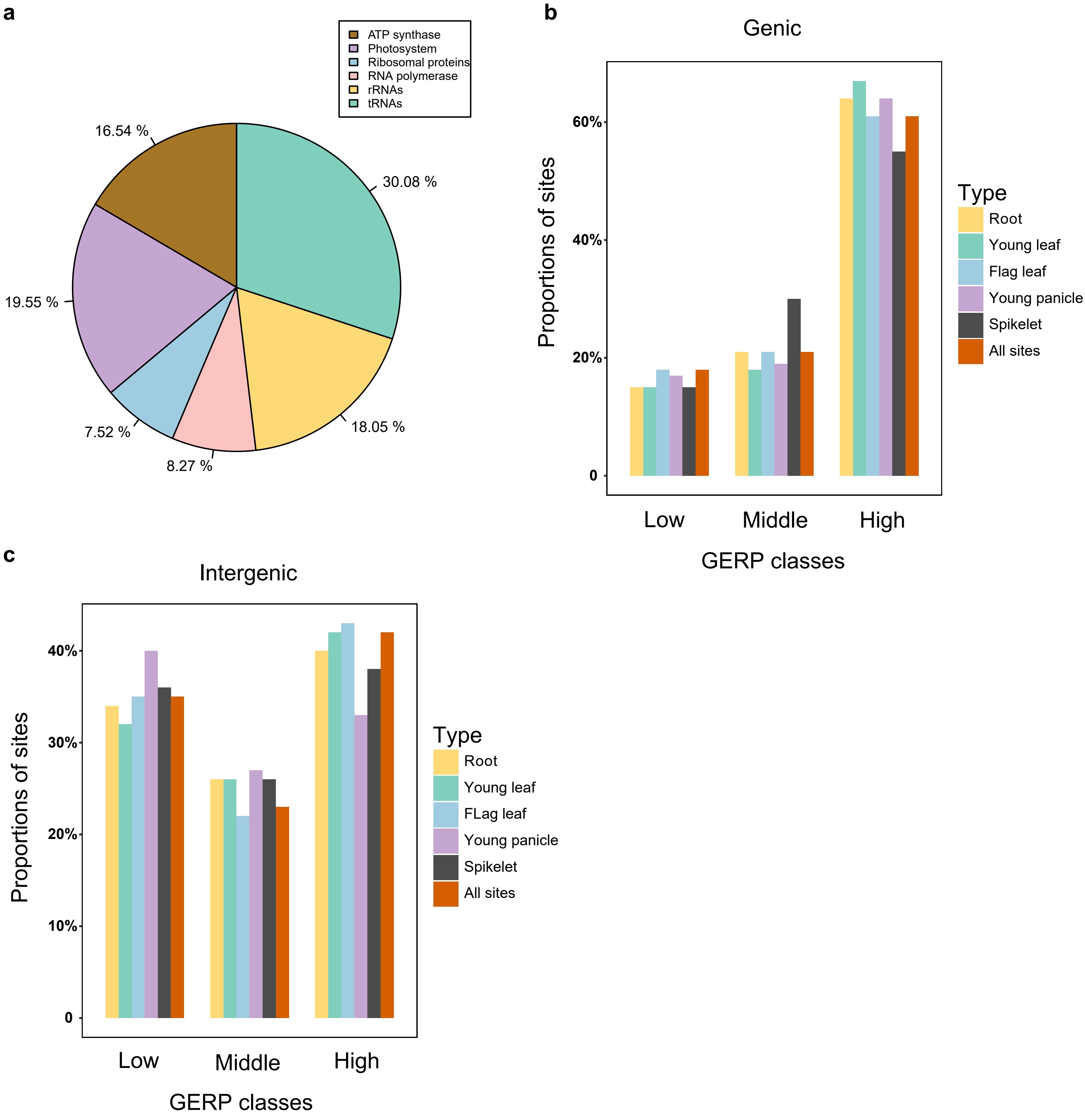

Supplementary Figure 6. Conservative analysis of CPAFPs. (a) Distribution of 133 C4-specific conserved CPAFPs by functional classifications of the genes in which they are located. The gene annotation of sorghum cpDNA was used. **(b)** The proportions of site numbers for CPAFPs located in genic regions in different GERP classes among five tissues identified in rice. The GERP classes are categories as “Low”, “Middle”, “High” according to the conservativeness scores (Methods). For each tissue, the proportion was calculated by dividing the site numbers in this class by the total site numbers of CPAFPs in the genic regions. **(c)** The proportions of site numbers for CPAFPs located in intergenic regions in different GERP classes among five tissues identified in rice. Details were same as **b**.

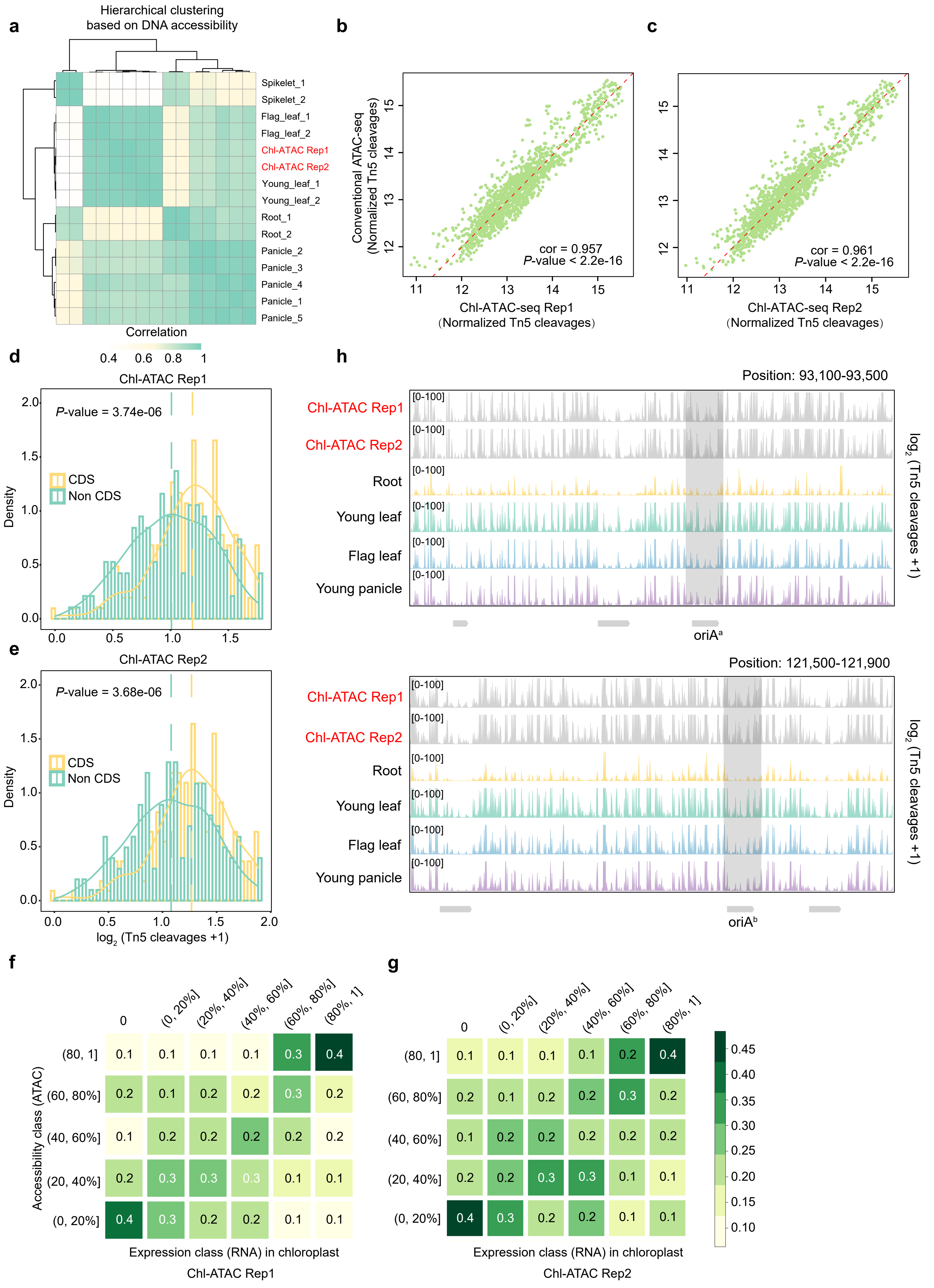

a
Hierarchical clustering based on DNA accessibility
Spikelet_1
Spikelet_2
Flag_leaf_1
Flag_leaf_2
Chl-ATAC Rep1
Chl-ATAC Rep2
Young_leaf_1
Young_leaf_2
Root_1
Root_2
Panicle_2
Panicle_3
Panicle_4
Panicle_1
Panicle_5
Correlation
0.4 0.6 0.8 1
b
Conventional ATAC-seq (Normalized Tn5 cleavages)
cor = 0.957
P-value < 2.2e-16
Chl-ATAC-seq Rep1 (Normalized Tn5 cleavages)
c
cor = 0.961
P-value < 2.2e-16
Chl-ATAC-seq Rep2 (Normalized Tn5 cleavages)
d
Chl-ATAC Rep1
P-value = 3.74e-06
CDS
Non CDS
Density
e
Chl-ATAC Rep2
P-value = 3.68e-06
CDS
Non CDS
Density
log2 (Tn5 cleavages +1)
h
Position: 93,100-93,500
Chl-ATAC Rep1
Chl-ATAC Rep2
Root
Young leaf
Flag leaf
Young panicle
[0-100]
oriA^a
log2 (Tn5 cleavages +1)
Position: 121,500-121,900
oriA^b
f
0
(0, 20%]
(20%, 40%]
(40%, 60%]
(60%, 80%]
(80%, 1]
(80, 1]
(60, 80%]
(40, 60%]
(20, 40%]
(0, 20%]
Accessibility class (ATAC)
Expression class (RNA) in chloroplast
Chl-ATAC Rep1
g
Expression class (RNA) in chloroplast
Chl-ATAC Rep2

Supplementary Figure 7. **(a)** Heatmap of correlations among chl-ATAC data of young leaves and conventional ATAC-seq data from different tissues. We divided the cpDNA into 100 bp bins and quantified the number of Tn5 insertions per bin, and then calculated the Spearman correlation coefficient between different datasets. **(b-c)**, The correlation between the two replicates of chl-ATAC data for young leaves with conventional ATAC-seq data for young leaves. Each point represents the number of Tn5 insertions in a 100 bp bin. **(d-e)**, The distributions of accessibility in genic and intergenic regions. The x-axis is average Tn5 cleavages of per region (first sum the Tn5 cleavages for each genic and intergenic regions, then divided by its length), while y-axis represents the density. **(f-g)**, The distribution of accessibility of regions with different expression signals in cpDNA. In the heatmap, the columns represent different levels of RNA-seq coverage and the rows represent different levels of Tn5 signal of ATAC-seq. The numbers in the cell represent the proportion of regions with a certain level of expression that have a certain level of accessibility, for example, the upper-right cell indicates that 40% of the top 20% high expression regions have their accessibility at the top 20% level. **(h)**, Profiles of Tn5 insertions around the two replication initiation sites, oriA, in multiple tissues of rice.

Supplementary Table 1. Summary of ATAC-seq data used in this study

Sample	Raw reads number	nDNA mapping reads number	nDNA mapping rate	cpDNA mapping reads number	cpDNA mapping rate	cpDNA average coverage
O.sativa_flag_leaf_1	92,533,216	90,913,885	98.25%	1,278,168	1.38%	1338.06
O.sativa_flag_leaf_2	163,670,488	158,122,058	96.61%	2,475,992	1.51%	2577.56
O.sativa_spikelet_1	101,666,618	99,958,619	98.32%	81,761	0.08%	84.70
O.sativa_spikelet_2	98,606,866	97,206,649	98.58%	85,009	0.09%	88.20
O.sativa_panicle_1	36,435,606	35,867,211	98.44%	280,272	0.77%	255.54
O.sativa_panicle_2	57,332,524	56,770,665	99.02%	268,979	0.47%	257.77
O.sativa_panicle_3	65,002,916	64,177,379	98.73%	448,164	0.69%	428.81
O.sativa_panicle_4	166,991,452	163,334,339	97.81%	854,849	0.51%	908.54
O.sativa_panicle_5	74,643,946	73,524,287	98.50%	881,459	1.18%	936.67
O.sativa_root_1	174,433,758	170,160,131	97.55%	640,647	0.37%	587.34
O.sativa_root_2	28,235,594	27,792,295	98.43%	250,534	0.89%	231.94
O.sativa_young_leaf_1	92,533,216	90,913,885	98.25%	5,974,672	6.46%	4100.16
O.sativa_young_leaf_2	163,670,488	158,122,058	96.61%	675,448	0.41%	640.13
B.distachyon_flag_leaf_1	94,067,702	91,198,637	96.95%	3,990,290	4.24%	3619.60
B.distachyon_flag_leaf_2	63,932,496	61,413,556	96.06%	2,855,203	4.47%	2743.20
B.distachyon_spikelet_1	39,572,726	37,736,552	95.36%	1,547,704	3.91%	1367.68
B.distachyon_spikelet_2	24,329,952	23,329,991	95.89%	878,194	3.61%	774.33
B.distachyon_panicle_1	116,923,382	113,602,758	97.16%	4,067,311	3.48%	3163.89
B.distachyon_root_1	14,543,028	6,857,038	47.15%	36,200	0.25%	26.00
B.distachyon_root_2	45,701,156	23,143,065	50.64%	112,352	0.25%	81.54
B.distachyon_young_leaf_1	10,870,214	10,439,754	96.04%	423,764	3.90%	361.76

Sample	Raw reads	Reads mapped to nDNA	nDNA mapping rate	Reads mapped to cpDNA	cpDNA mapping rate	cpDNA average coverage
B.distachyon_young_leaf_2	10,291,646	10,038,472	97.54%	348,198	3.38%	295.76
S.italica_flag_leaf_1	141,996,604	139,611,061	98.32%	15,857,596	11.17%	7589.89
S.italica_flag_leaf_2	100,706,220	99,850,217	99.15%	12,752,313	12.65%	7401.39
S.italica_spikelet_1	131,086,536	129,972,300	99.15%	2,914,716	2.22%	2861.65
S.italica_spikelet_2	138,357,024	137,333,182	99.26%	3,522,005	2.55%	3455.01
S.italica_panicle_1	177,674,200	176,323,876	99.24%	7,139,033	4.02%	6175.56
S.italica_panicle_2	150,142,666	148,941,525	99.20%	8,660,612	5.77%	6774.23
S.italica_root_1	25,735,750	25,434,642	98.83%	419,956	1.63%	154.9
S.italica_young_leaf_1	70,067,138	62,794,169	89.62%	1,738,689	2.48%	638.256
S.italica_young_leaf_2	57,397,792	55,641,420	96.94%	6,374,331	11.1%	5454.16
S.bicolor_flag_leaf_1	130,334,958	129,383,513	99.27%	3,107,500	2.38%	3052.2
S.bicolor_flag_leaf_2	144,450,460	143,309,301	99.21%	3,934,117	2.72%	3757.47
S.bicolor_spikelet_1	203,189,918	202,316,201	99.57%	1,090,657	0.54%	1028.62
S.bicolor_spikelet_2	245,343,676	244,509,508	99.66%	853,513	0.35%	779.83
S.bicolor_panicle_1	84,595,194	83,072,481	98.20%	7,044,379	8.33%	6259.61
S.bicolor_panicle_2	182,882,314	180,504,844	98.70%	15,983,068	8.74%	7624.41
S.bicolor_root_1	63,639,878	55,544,886	87.28%	8,653,897	13.60%	3031.31
S.bicolor_root_2	397,222,962	393,528,788	99.07%	14,229,377	3.58%	7403.26
S.bicolor_young_leaf_1	112,887,230	108,507,205	96.12%	32,749,089	29.01%	6791.13
S.bicolor_young_leaf_2	8,595,822	8,488,374	98.75%	1,490,993	17.35%	1463.3
Z.mays_ear_1	92,636,560	92,266,014	99.60%	8,325,168	8.99%	6511.58
Z.mays_ear_2	96,513,184	95,924,454	99.39%	9,305,203	9.64%	6768.54
Z.mays_flag_leaf_1	95,249,354	94,954,081	99.69%	1,588,152	1.67%	1223.37

Sample	Raw reads	Reads mapped to nDNA	nDNA mapping rate	Reads mapped to cpDNA	cpDNA mapping rate	cpDNA average coverage
Z.mays_flag_leaf_2	100,750,444	100,256,767	99.51%	2,488,664	2.47%	1728.09
Z.mays_spikelet_1	105,904,206	105,088,744	99.23%	516,052	0.49%	422.19
Z.mays_spikelet_2	112,607,990	112,326,470	99.75%	552,991	0.49%	440.24
Z.mays_root_1	226,773,650	113,931,082	50.24%	5,245,362	2.31%	1851.97
Z.mays_root_2	158,419,674	68,437,299	43.20%	3,522,866	2.32%	1242.55
Z.mays_root_3	159,349,756	158,951,382	99.75%	2,918,492	1.83%	2505.56
Z.mays_young_leaf_1	141,333,474	134,591,867	95.23%	28,981,883	20.51%	6462.91
Z.mays_young_leaf_2	103,976,508	98,611,320	94.84%	17,515,251	16.85%	5025.53
Z.mays_young_leaf_3	126,330,162	122,881,349	97.27%	1,573,734	1.25%	1195.25

Supplementary Table 2. Summary of RNA-seq data used in this study

Sample	Raw reads	Reads mapped to nDNA	nDNA mapping rate	Reads mapped to cpDNA	cpDNA mapping rate	Sequencing depth of cpDNA
ZS97_young_leaf	85,814,070	34,325,628	99.15%	482,163	0.56%	493.22
ZS97_root	75,153,831	30,061,532	99.26%	25,482	0.03%	30.04

Supplementary Table 3. Summary of CPAFP information in different tissues across species

Species	Tissue	Reads mapped to cpDNA	Average coverage of cpDNA	CPAFP number	Total length of CPAFP
O.sativa	Root	891,181	993.69	391	8,605
	Young Leaf	6,650,120	7415.11	413	9,740
	Flag Leaf	3,754,160	4186.02	431	10,054
	Young Panicle	2,733,723	3048.19	412	9,453
	Spikelet	166,770	185.95	336	7,945
B.distachyon	Root	148,552	164.81	None	None
	Young Leaf	771,962	856.47	308	6,762
	Flag Leaf	6,845,493	7594.90	453	9,927
	Young Panicle	4,067,311	4512.58	357	7,637
	Spikelet	2,425,898	2671.47	218	4,404
S.italica	Root	419,956	466.04	263	5,765
	Young Leaf	8,113,020	5768.15	388	8,562
	Flag Leaf	28,609,909	31656.0	428	9,218
	Young Panicle	15,799,645	17481.86	429	9,345
	Spikelet	6,436,721	7122.05	406	8,892
S.bicolor	Root	22,883,274	24386.45	751	16,617
	Young Leaf	34,240,082	36489.28	865	18,559
	Flag Leaf	7,041,617	7504.17	548	12,196
	Young Panicle	23,027,447	24540.09	649	14,639
	Spikelet	1,944,170	2071.88	430	9,448
Z.mays	Root	11,686,720	12487.23	702	15,776
	Young Leaf	48,070,868	51363.62	734	15,940
	Flag Leaf	4,076,816	4356.07	405	9,013
	Ear	17,630,371	18838.01	685	14,495
	Spikelet	1,069,043	1142.27	405	9,229

Supplementary Table 4. Fisher's exact test of the enrichment of 4DS in conserved footprints

	Non-overlap with conserved footprint	Overlap with conserved footprint	Total
4DS	9,459	81	9,540
Non-4DS	17,908	819	18,727
Total	27,367	900	28,267
		**OR = 5.34; *P*-value < 2.2e-16**	

Supplementary Table 5. Paired-*t*-test of accessibility in CPAFPs-flanking regions between leaves and roots

Gene functions closest to CPAFPs	Group1	Group2	CPAFP numbers	p.adj	p.adj.signif
ATP	Flag leaf	Root	75	0.128	ns
ATP	Flag leaf	Young leaf	75	0.000021	***
ATP	Root	Young leaf	75	0.00023	***
Other proteins	Flag leaf	Root	27	0.178	ns
Other proteins	Flag leaf	Young leaf	27	0.098	ns
Other proteins	Root	Young leaf	27	1	ns
photosystem	Flag leaf	Root	70	1.52E-05	****
photosystem	Flag leaf	Young leaf	70	1	ns
photosystem	Root	Young leaf	70	1.21E-06	****
ribosomal proteins	Flag leaf	Root	27	0.22	*
ribosomal proteins	Flag leaf	Young leaf	27	1	ns
ribosomal proteins	Root	Young leaf	27	0.05	ns
RNA polymerase	Flag leaf	Root	74	1	ns
RNA polymerase	Flag leaf	Young leaf	74	5.19E-16	****
RNA polymerase	Root	Young leaf	74	7.11E-12	****
rRNA	Flag leaf	Root	97	6.27E-07	****
rRNA	Flag leaf	Young leaf	97	2.14E-07	****
rRNA	Root	Young leaf	97	0.071	ns
tRNA	Flag leaf	Root	51	0.004	**
tRNA	Flag leaf	Young leaf	51	0.002	**
tRNA	Root	Young leaf	51	0.049	*

Supplementary Table 6. Paired-*t*-test of FPD between leaves and roots

Gene functions closest to CPAFPs	Group1	Group2	CPAFP numbers	p.adj	p.adj.signif
ATP	Flag leaf	Root	75	2.68E-14	****
ATP	Flag leaf	Young leaf	75	0.0000363	****
ATP	Root	Young leaf	75	5.91E-07	****
Other proteins	Flag leaf	Root	27	0.073	ns
Other proteins	Flag leaf	Young leaf	27	1	ns
Other proteins	Root	Young leaf	27	0.017	*
Photosystem	Flag leaf	Root	70	5.82E-13	****
Photosystem	Flag leaf	Young leaf	70	0.000768	***
Photosystem	Root	Young leaf	70	3.81E-08	****
Ribosomal proteins	Flag leaf	Root	27	3.15E-05	****
Ribosomal proteins	Flag leaf	Young leaf	27	0.263	ns
Ribosomal proteins	Root	Young leaf	27	0.008	**
RNA polymerase	Flag leaf	Root	74	1.02E-15	****
RNA polymerase	Flag leaf	Young leaf	74	8.67E-09	****
RNA polymerase	Root	Young leaf	74	4.68E-06	****
rRNA	Flag leaf	Root	97	1.35E-11	****
rRNA	Flag leaf	Young leaf	97	1.75E-09	****
rRNA	Root	Young leaf	97	3.75E-22	****
tRNA	Flag leaf	Root	51	8.01E-06	****
tRNA	Flag leaf	Young leaf	51	0.000456	***
tRNA	Root	Young leaf	51	2.03E-11	****

Supplementary Table 7. Subject CPAFPs: CPAFPs identified in young leaves

No. sites (Prop.) / GERP class	Genic		Intergenic	
	Subject-CPAFPs	Others	Subject-CPAFPs	Others
Low	1,100 (15%)	13,561 (18%)	947 (32%)	17,504 (35%)
Middle	1,212 (18%)	15,823 (21%)	773 (26%)	11,352 (23%)
High	4,510 (67%)	45,623 (61%)	1,230 (42%)	20,970 (42%)
Z-test for proportions	*P*-value = 4.81e-17		*P*-value = 2.0e-05	

Supplementary Table 8. Subject CPAFPs: CPAFPs identified in all five species

No. sites (Prop.) / GERP class	Genic		Intergenic	
	Subject-CPAFPs	Others	Subject-CPAFPs	Others
Low	149 (7%)	14,424 (18%)	42 (20%)	18,419 (35%)
Middle	102 (4%)	16,961 (21%)	3 (1%)	12,122 (23%)
High	2,015 (89%)	48,088 (61%)	169 (79%)	22,031 (42%)
Z-test for proportions	*P*-value = 1.1e-164		*P*-value = 1.95e-28	
