# Supplementary material for: Characterization of the DNA accessibility of chloroplast genomes in grasses
Source: Commun Biol. 2024 Jun 22;7:760. doi: 10.1038/s42003-024-06374-4 (PMC11193712; doi:10.1038/s42003-024-06374-4)
Supplement: Supplementary file 3 — Description of Additional Supplementary Files [file 42003_2024_6374_MOESM3_ESM.pdf]

Description of Additional Supplementary Files

File name: **Supplementary Data 1**

Description: The source data behind the graphs in the paper of Figure 1

File name: **Supplementary Data 2-5**

Description: The source data behind the graphs in the paper of Supplementary Figure 4

File name: **Supplementary Data 6-8**

Description: The source data behind the graphs in the paper of Figure 5
